# Absence of Common Somatic Alterations in Genes on 1p and 19q in Oligodendrogliomas

**DOI:** 10.1371/journal.pone.0022000

**Published:** 2011-07-07

**Authors:** Linda B. Bralten, Stephan Nouwens, Christel Kockx, Lale Erdem, Casper C. Hoogenraad, Johan M. Kros, Michael J. Moorhouse, Peter A. Sillevis Smitt, Peter van der Spek, Wilfred van Ijcken, Andrew Stubbs, Pim J. French

**Affiliations:** 1 Department of Neurology, Erasmus Medical Center, Rotterdam, The Netherlands; 2 Department of Bioinformatics, Erasmus Medical Center, Rotterdam, The Netherlands; 3 Center for Biomics, Erasmus Medical Center, Rotterdam, The Netherlands; 4 Department of Neuroscience, Erasmus Medical Center, Rotterdam, The Netherlands; 5 Department of Pathology, Erasmus Medical Center, Rotterdam, The Netherlands; University of Chicago, United States of America

## Abstract

A common and histologically well defined subtype of glioma are the oligodendroglial brain tumors. Approximately 70% of all oligodendrogliomas have a combined loss of the entire 1p and 19q chromosomal arms. This remarkably high frequency suggests that the remaining arms harbor yet to be identified tumor suppressor genes. Identification of these causal genetic changes in oligodendrogliomas is important because they form direct targets for treatment. In this study we therefore performed targeted resequencing of all exons, microRNAs, splice sites and promoter regions residing on 1p and 19q on 7 oligodendrogliomas and 4 matched controls. Only one missense mutation was identified in a single sample in the *ARHGEF16* gene. This mutation lies within- and disrupts the conserved PDZ binding domain. No similar ARHGEF16 mutations or deletions were found in a larger set of oligodendrogliomas. The absence of common somatic changes within genes located on 1p and 19q in three out of four samples indicates that no additional “second hit” is required to drive oncogenic transformation on either chromosomal arm.

## Introduction

A common and histologically well defined subtype of glioma are the oligodendroglial brain tumors. Oligodendrogliomas differ from the other glioma subtypes in clinical behavior with respect to overall prognosis (median survival 3 years versus less than one year) and a relatively better and longer lived response to chemotherapy and radiotherapy [1]–[3]. Oligodendrogliomas have clearly distinct gene expression profiles [4]–[6] and are also cytogenetically distinct: approximately 70% of all oligodendrogliomas have a combined loss of the entire short arm of chromosome 1 (1p) and loss of the entire long arm of chromosome 19 (19q) [1], [3]–[5], [7]. Loss of these chromosomal arms in oligodendrogliomas is highly correlated with chemosensitivity; approximately 80–90% of oligodendroglial tumors with LOH (loss of heterozygosity) on 1p and 19q respond to chemotherapy [1], [2], [8]. Conversely only 25–30% of tumors that have retained the short arm of chromosome 1p are sensitive to chemotherapy. In summary, oligdendrogliomas are a clinically, histologically, cytogenetically and molecularly distinct and well defined subgroup of glioma.

In spite of these clearly distinct clinical, histological and molecular features, little is known on the genetic changes that drive these tumors. Thusfar, IDH1/IDH2 (70%) and, to a much lesser extent, TP53 (15–25%) and PIK3CA (10–15%) are the only genes that are mutated at significant frequency in this tumor type [9]–[15]. The remarkably high frequency of LOH of 1p and 19q suggests the remaining arms harbor yet to be identified tumor suppressor genes (Knudson two-hit hypothesis [16]). Identification of the causal genetic changes is important because they form direct targets for treatment: Tumor growth depends on these acquired-somatic- changes both in oncogenes (“oncogene addiction” [17]) and in tumor suppressor genes [18], [19]. In this study we therefore aimed to identify genetic changes in all exons, microRNAs, splice sites and promoter regions on 1p or 19q using array capture and Next Generation Sequencing. Experiments were performed on 7 oligodendrogliomas and 4 had matched control DNA samples.

## Materials and Methods

Glioma samples were collected from the Erasmus MC tumor archive. Samples were collected immediately after surgical resection, snap frozen, and stored at −80°C. The use of patient material was approved of by the Institutional Review Board of the Erasmus MC, Rotterdam, the Netherlands (nr MEC 221.520/2002/262; date of approval July 22, 2003, and MEC-2005-057, date of approval February 14, 2005). For this use, patients gave written informed consent according to institutional and national guidelines.

All oligodendrogliomas used (n = 7) had proven loss of 1p and 19q as assessed by SNP 6.0 or 250 k *NspI* arrays (both Affymetrix, Santa Clara, USA) [7] and highly similar RNA expression profiles (i.e. belong to the same molecular subgroup) [6]. Control DNA was available in 4/7 cases. The candidate variations of 4 samples were used for validation experiments. By using 4 samples we have a 76.0% chance of identifying each mutation with a frequency of 30%. DNA was amplified using a Repli-G midi kit (Qiagen, Venlo, the Netherlands) to ensure sufficient DNA amounts. Patient characteristics are listed in table 1.

**Table 1:** Patient characteristics of all tumor samples.

Sample	Gender	Diagnosis	Age	KPS	Surgery	RT	CT	Alive	Surv (years)
**8**	F	OD III	44	100	PR	yes	no	Dead	9.82
**11**	M	OD III	38		CR	yes	no	Dead	8.92
**13**	M	OD III	33	90	PR			Dead	8.59
**21**	M	OD III	31	100	PR	yes	no	Dead	6.81
**23**	F	OD III	44	90	CR	yes	no	Dead	8.12
**229**	M	OA III	35	90	CR	yes	Adj PCV	Alive	6.8
**538**	F	OD II	44	80	SB			Alive	3.27

OD  =  oligodendroglioma, OA  =  oligoastrocytoma, KPS  =  Karnofsky performance score,

PCV  =  procarbazine, lomustine, vincristine.

F =  female, M =  male, OD  =  oligodendroglioma, OA  =  oligoastrocytoma, grades II or III. Age =  age at diagnosis. KPS  =  Karnofsky performance score, PCV  =  procarbazine, lomustine, vincristine. Surgery types: PR =  partial resection, CR =  complete resection, SB =  stereotactic biopsy. RT =  radiotherapy, CT =  Chemotherapy.

Capture arrays (Nimblegen, Roche NimbleGen, Inc., Waldkraiburg, Germany) were designed to enrich for all exons, miRNAs, splice sites (defined as 10 bp up- or downstream of a coding exon) and promoter regions (defined as 100 bp upstream of a transcript) of transcripts present in Refseq, Ensembl or Vega based on the NCBI36/hg18 build. Two capture arrays were designed covering around 5 million bp of sequence each. We were able to design capture probes for 96.0% and 94.4% of all regions on chr 1 and chr 19 respectively, remaining sequences contained non unique sequences (>5 fold presence in the human genome). Amplified DNA samples were fragmented by sonication, end repaired and ligated to paired end adaptors. Samples were size selected (300 bp), enriched for 1p and 19q by array capture, PCR-amplified and 76 bp paired end sequenced using the Illumina GA2x sequencer. The Illumine Casava pipeline was used for base calling and quality control.

The CLC Bio Genomics Workbench (Aarhus, Denmark) was used to align sequence reads against the reference genome. All the experiments were successful except for one of the two capture arrays of sample 229, which was omitted from the analysis. We defined reads subject for mutational analysis as being covered at least 7 times in the tumor sample and at least 8 times in the matching control sample. This coverage was set deliberately low (often ≥30 fold coverage is used) and was aimed to include as many evaluable targeted regions as possible.

Single Nucleotide Variants (SNVs) and Deletion or Insertion Variants (DIVs) were detected by the CLC Bio Genomic Workbench and filtered using the following criteria (coverage of at least 7 (tumor) or 8 (ctr), variant frequency at least 70% (tumor) or 30% (controls). The difference in variant frequency between tumors and normals is because only homozygous changes are expected in the tumor (because of the 1p19q LOH). To allow for tumor heterogeneity (presence of non-neoplastic tissue) and stochastic effects (allele specific sequencing) these percentages were set lower than 100% and 50%.

## Results

**Figure 1:**
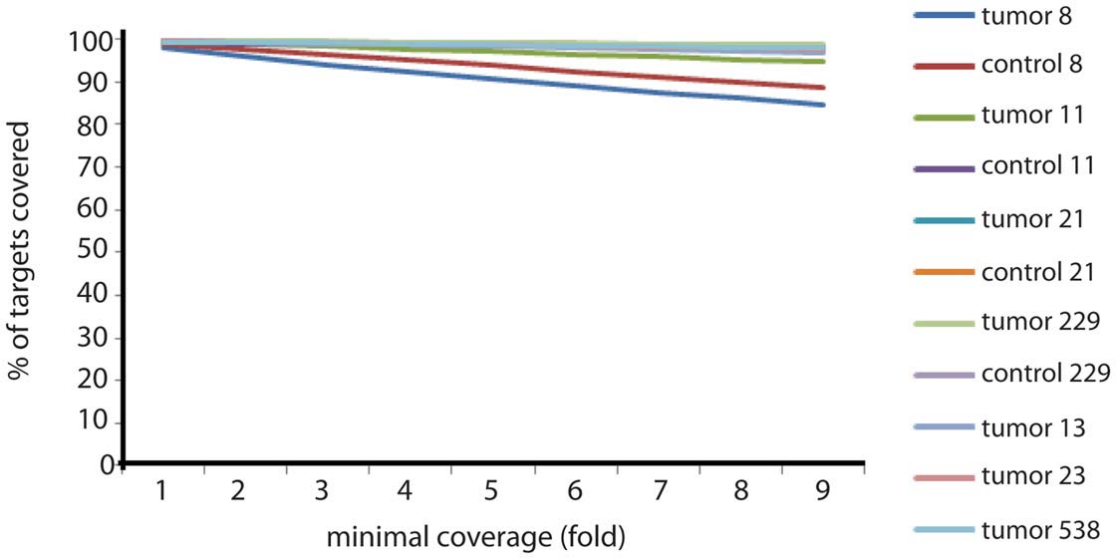
Coverage plot of all samples. Depicted is the percentage of targeted bases (y-axis) that is covered at least n times (x-axis) per sample.

The mean total number of matched bp sequenced per sample was 2.89 billion (1.17 billion in sample 229). Of these, 73.5% was on our targeted regions (range 70.4–84.8%, one outlier at 41.3%) confirming capture efficiency. The coverage of our targeted regions was at least 7 in 96.5% of our target regions (range 87.6–98.9) (see also figure 1). Coverage of genes suggested to be involved in (oligodendro-) glioma genesis CAMTA1 [20], EMP3 [21], CHD5 [22], DIRAS3 [23] and PLA2G4C [24] is listed in table 2.

**Table 2:** Per base coverage of known candidate genes in oligodendrogliomas located on 1p or 19q.

Sample		CAMTA	CHD5	DIRAS3	PLA2G4C	EMP3
**8**	*cov*	200.1	264.4	107.2	32.3	57.0
	*min*	0	5	26	2	15
	*max*	896	1198	186	87	131
	*% covered*	96.2	99.6	100	93.1	100
						
**11**	*cov*	58.6	20.2	127.0	79.0	90.2
	*min*	0	0	12	7	33
	*max*	275	107	231	214	183
	*% covered*	93.5	82.1	100	100	100
						
**13**	*cov*	184.6	135.8	98.5	183.8	343.1
	*min*	0	0	14	24	126
	*max*	562	677	178	447	625
	*% covered*	98.2	94.0	100	100	100
						
**21**	*cov*	163.8	77.7	208.1	202.8	281.3
	*min*	0	2	24	50	95
	*max*	527	329	360	449	599
	*% covered*	96.8	98.9	100	100	100
						
**23**	*cov*	178.8	69.0	245.8	189.4	202.3
	*min*	0	0	35	21	70
	*max*	531	353	468	487	374
	*% covered*	95.9	89.9	100	100	100

Min/Max: lowest/highest coverage, % covered: the percentage of bases sequenced at least 7 times (the cutoff used for our analysis).

We first calculated the tumor percentage of all samples. The tumor content can be estimated by the observed B allele frequency of SNPs using tumor samples only. For example, in case of 50% tumor, the observed B allele frequency would amount to 66.7% (in case of LOH in the tumor). In our sample cohort, the observed B allele frequency was 91.2–97.3% (except for sample 11 with an observed frequency of 85.0%), corresponding to 90.3–97.2% tumor (82.4% for sample 11). A tumor content ≥82.4% indicates that a mutant allele frequency of 70% (corresponding to a tumor percentage of 82.4%) is a suitable value as detection cutoff for the mutant allele frequency.

We then prioritized all changes identified based on their associated function into: tier 1 (coding exons, splice sites and miRNAs), tier 2 (promoters and UTRs), tier 3 (intronic regions) and tier 4 (SNPs and personal SNPs). In tiers 1, 2, 3 and 4 we identified 431, 1380, 16293 and 72466 SNVs and DIVs respectively. A high number of all variants present in tier 4 were not present in dbSNP130, and are likely to reflect personal SNPs and sequencing artefacts (see also table 3). We then performed direct sequencing on all tier 1 candidates and all candidates within promoter regions (part of tier 2). 505 of the 514 sequence reactions were successful; only 2/9 unsuccessful candidates were predicted to result in a change in the primary protein sequence (both missense mutations).

**Table 3:** Candidate genetic variations after filtering in all samples with controls.

SNVs			tier 1			tier 2		tier 3	tier 4	
sample	*array*	*chr*	*exons*	*miRNA*	*spl site*	*prom*	*UTRs*	*introns*	*SNVs dbSNP*	*personal SNVs*
8	1	1	13	0	4	9	96	97	4312	1577
	2	1	33	0	3	3	218	38	1020	841
	2	19	19	0	0	6	37	23	1350	1120
11	1	1	80	0	3	4	110	68	3342	852
	2	1	14	0	2	5	110	204	1787	3400
	2	19	108	0	1	3	59	369	2703	4146
21	1	1	17	0	2	4	92	891	4450	1095
	2	1	13	0	0	7	94	3166	2110	7892
	2	19	25	0	0	4	63	3887	3090	9028
229	2	1	20	0	3	9	144	3505	146	3887
	2	19	44	0	2	5	135	3543	287	13213

DIVs			tier 1			tier 2		tier 3	tier 4	
sample	*array*	*chr*	*exons*	*miRNA*	*spl site*	*prom*	*UTRs*	*introns*	*DIVs dbSNP*	personal DIVs
**8**	1	1	0	0	10	11	60	79	179	0
	2	1	0	0	2	1	6	3	18	0
	2	19	1	0	0	1	9	7	54	0
**11**	1	1	0	0	6	6	27	51	118	1
	2	1	0	0	0	0	1	0	1	0
	2	19	0	0	0	0	0	0	0	0
**21**	1	1	0	0	0	0	0	0	0	0
	2	1	0	0	0	0	0	1	0	0
	2	19	0	0	0	0	0	0	0	0
**229**	2	1	2	0	0	5	24	134	181	28
	2	19	3	0	1	0	12	227	177	61

UTR =  untranslated region, SNV =  single nucleotide variation, DIV =  deletion/insertion variation, chr = chromosome, array =  capture array, spl site =  splice site, prom = promoter.

**Figure 2:**
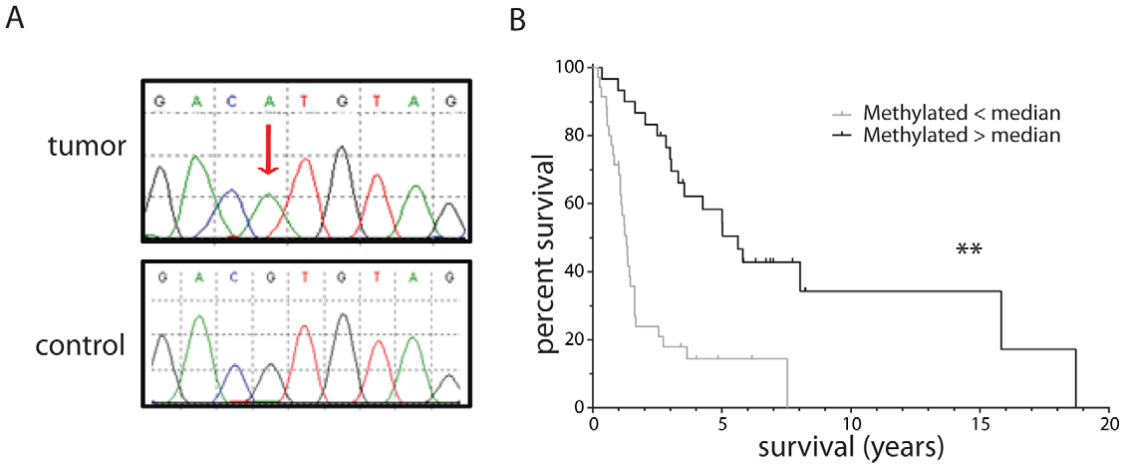
ARHGEF16 (RefSeq: NM_014448.3) mutation and promoter methylation. A; Upper lane: part of the sequence of ARHGEF16 with the missense mutation (2125G->A) in tumor sample 8. Lower lane: sequence of the same region of ARHGEF16 in the matching control DNA. B; Kaplan Meier survival curve of oligodendrogliomas (n = 39) and oligoastrocytomas (n = 11) with unmethylated ARHGEF16 (< median) (black line) or methylated ARHGEF16 (> median) (grey line). ** = p<0.01.

Of the 514 candidate variants 77% (n = 394) were not confirmed on tumor DNA using direct sequencing (false positive). Such variants likely represent amplification artefacts (due to e.g. whole genome amplification or the post capture PCR amplification) and/or sequencing artefacts (e.g. sequencing errors). A further 21% (n = 110) could be confirmed in the tumor samples, but the variant was also present in the matched control DNA. These variants may represent selective allele amplification and sequencing. In summary, of the 514 candidates subject to direct sequencing, one variant was validated. This variant is a missense mutation (c.2125 G>A) (figure 2a) and affects the last amino acid (p.V709M) of ARHGEF16 (RefSeq: NM_014448.3) in sample 8. It should be noted that the absence of trace wt sequence in the chromatogram confirms the high tumor percentage in this sample. The base is highly conserved (GERP conservation score 3.35 [25]. This amino acid is located within a PDZ-binding domain (ETDV, a protein-protein interaction domain). However, it remains to be determined whether the identified mutation affects its RhoA guanine exchange function and oncogenic transformation potential [26].

None of the other 6 samples contained changes in the coding sequence of ARHGEF16. In addition, we failed to identify mutations in the last exon of ARHGEF16 in an additional 32 samples from the same molecular cluster [6] using direct sequencing. No small homozygous deletions were identified on SNP 6.0 and 250 k Nsp arrays from 23 oligodendrogliomas [7]. The ARHGEF16 promoter does show hypermethylation on Infinium Methylation arrays (Illumina, San Diego, USA), on 68 anaplastic oligodendrogliomas and oligoastrocytomas; PF, manuscript in prep) and is correlated with loss of 1p and 19q (p = 0.035, Fisher exact test). Data are listed in table 4. In addition, tumors with hypermethylated ARHGEF16 promoter have a better survival (5.62 years versus 1.31 years; p<0.0001) (figure 2b). Promoter methylation of ARHGEF16 may therefore be involved in the formation of gliomas with loss of 1p and 19q.

**Table 4:** Percentage methylation of two different CpG sites (cg24919884 and cg02737335) within the ARHGEF16 locus.

	ARHGEF16								
Sample	cg24919884	cg02737335	survival	censoring	Sample	cg24919884	cg02737335	survival	censoring
1	0.767	0.69	1.104	1	42	0.874	0.817	1.633	1
10	0.642	0.754	1.071	1	43	0.691	0.794	1.208	1
11	0.777	0.829	4.008	0	44	0.788	0.739	1.063	1
12	0.657	0.285	3.408	0	45	0.879	0.845	7.734	0
13	0.251	0.313	2.548	1	47	0.565	0.663	1.31	1
14	0.828	0.831	0.775	1	48	0.81	0.821	0.523	1
15	0.773	0.771	0.326	1	49	0.876	0.84	5.003	1
16	0.565	0.737	1.663	1	5	0.617	0.813	2.732	1
17	0.867	0.872	3.54	1	50	0.623	0.8	0.195	1
18	0.487	0.609	1.416	1	51	0.866	0.802	5.814	1
19	0.706	0.688	0.819	1	52	0.855	0.834	8.227	0
20	0.681	0.688	1.625	1	53	0.862	0.835	2.997	1
21	0.851	0.823	2.485	1	54	0.439	0.469	1.34	1
22	0.878	0.829	2.627	0	55	0.879	0.824	8.036	1
23	0.835	0.822	0.625	1	56	0.852	0.831	8.233	0
24	0.857	0.849	5.827	0	57	0.704	0.4	0.707	1
25	0.412	0.729	1.625	1	58	0.836	0.778	0.975	1
26	0.615	0.646	1.447	1	59	0.846	0.824	2.022	1
27	0.821	0.854	6.17	0	6	0.503	0.468	1.014	1
28	0.773	0.818	1.236	1	60	0.856	0.85	3.038	1
29	0.635	0.712	0.548	1	62	0.817	0.819	0.997	0
3	0.888	0.861	1.222	1	63	0.882	0.83	6.992	0
30	0.883	0.874	4.26	1	64	0.803	0.758	0.258	1
31	0.439	0.635	1.134	1	65	0.88	0.854	5.019	1
32	0.851	0.811	3.488	0	66	0.819	0.798	3.304	1
33	0.415	0.53	4.849	0	67	0.527	0.752	0.537	1
34	0.858	0.826	3.488	0	68	0.884	0.844	5.622	1
35	0.882	0.857	6.874	0	69	0.847	0.813	15.819	1
36	0.853	0.802	0.348	1	7	0.494	0.692	1.616	1
37	0.866	0.83	6.71	0	70	0.843	0.841	18.715	1
39	0.839	0.809	2.836	1	73	0.465	0.631	3.652	1
4	0.909	0.815	6.31	0	74	0.723	0.625	1.175	1
40	0.413	0.63	7.526	1	8	0.307	0.715	1.345	1
41	0.834	0.721	3.795	1	9	0.835	0.817	2.038	1

Values correspond to the fraction of methylation (scale 0–1).

## Discussion

We have systematically sequenced all exons, miRNAs, splice sites and promoter regions on 1p and 19q. Of the 514 candidate variants in coding exons, miRNAs, splice sites and promoter regions, only one was validated: a missense mutation in ARHGEF16 affecting the PDZ-binding domain. ARHGEF16 lies on 1p36 a region that is commonly deleted in gliomas [20], [22], [27]. However, no other genetic changes were detected in the ARHGEF16 gene in a panel of 32 additional oligodendrogliomas, though the promoter is frequently hypermethylated. Future experiments should determine whether this specific mutation contributes to the pathogenesis of the disease.

In our sample cohort, only one somatic mutation in a single sample was identified among the ∼10^7^ bases of sequence evaluated. The overall mutation rate in oligodendrogliomas therefore is at least an order of magnitude lower than reported for many other cancer types including glioblastomas [12], [28], [29]. Recent reports however, have highlighted tumor types that also have a very low somatic mutation rate [30], [31].

One important observation by our study is the fact that in 3 out of the four samples examined no genomic second hit was found in any of the screened regions. Our data therefore indicate that no additional “second hit” is required on these chromosomal arms to drive oncogenic transformation. It is possible that a second hit is present on the remaining alleles but has escaped detection (e.g. due to a skewed distribution in sequencing of the non-neoplastic alleles derived from “contaminating” normal tissue) or is located in regions not captured or covered by our capture array. Alternatively, promoter methylation and/or haploinsufficiency of one or more genes could drive oncogenesis in oligodendrogliomas with loss of 1p and 19q. In line with this hypothesis is the high frequency of IDH1 mutations in oligodendrogliomas with 1p and 19q LOH (see e.g. [32]) and the observation that IDH1 mutations induce chromatin remodeling and promoter hypermethylation [33], [34]. IDH1 mutations are associated with the CpG island hypermethylation phenotype (MvdB and PF, submitted), and promoter hypermethylation in cancer often occurs in the promoter regions of tumor suppressor genes [35], [36]. Promoter hypermethylation of tumor suppressor genes in combination with somatic mutations in a limited number of genes therefore may drive oncogenesis in oligodendrogliomas with 1p and 19q LOH.
